# Cocaine-Associated Skin-Picking and Severe Nasal Cleft Injury in a Patient With Trauma-Related Psychiatric Disorder: A Case Report

**DOI:** 10.1192/bjo.2026.11748

**Published:** 2026-06-30

**Authors:** Hannah Pasha Memon, Sridhar Shanmugham

**Affiliations:** hertfordshire partnership NHS foundation trust, Borehamwood, United Kingdom

## Abstract

**Aims:**

Compulsive skin-picking is a recognised complication of cocaine misuse, frequently driven by formication and somatic delusional beliefs of infestation.¹² Repetitive excoriation may result in significant tissue damage, impaired wound healing, and long-term disfigurement, particularly when combined with psychiatric comorbidity, poor insight, and psychosocial adversity. This case highlights the interaction between trauma-related psychopathology, cocaine-induced perceptual disturbance, and severe self-inflicted facial injury.

**Methods:**

LA, a 60-year-old woman from Brazil who moved to the UK over 20 years ago, presented with a history of trauma-related psychiatric symptoms and recurrent cocaine misuse. She had developed progressive self-inflicted injury to the nasal cleft and upper lip, particularly during periods of cocaine use. The formication and infestation beliefs also lead to compulsive aggressive cleaning of the philtrum and nasal region, including the use of sharp implements and a toothbrush, resulting in extensive soft-tissue damage and chronic non-healing wounds.

LA intermittently denied ongoing infestation beliefs but the picking behaviours continued, particularly during periods of medication non-adherence and substance use. Collateral history confirmed continued delusional beliefs. She was treated with Fluoxetine 60mg OM and Olanzapine 5mg ON. Dermatological assessment reported pyoderma-like ulceration, exacerbated by repeated trauma and cocaine adulterants. Despite periods of partial healing, recurrent excoriation prevented sustained recovery leading to consideration of future reconstructive surgery.

Management was complicated by inconsistent engagement, housing instability, and fluctuating insight. Escalation to depot antipsychotic therapy was considered due to ongoing self-injury and poor adherence. Psychological assessment identified significant functional impairment, compulsive behaviour, and substance misuse disorder, with reliance on family members for daily support.

**Results:**

Chronic intranasal cocaine use causes nasal and dermatological tissue damage through intense vasoconstriction, leading to ischaemia, ulceration, and tissue necrosis.³ Over time, this may progress to nasal septal perforation, collapse of nasal structures, and destruction of midline facial anatomy, including the philtrum and upper lip.

This pattern of injury is described as cocaine-induced midline destructive lesions (CIMDL), resulting from repeated vascular insult and progressive breakdown of nasal mucosa, cartilage, and surrounding soft tissues.^4^ Adulterants such as levamisole further contribute to vasculitic and necrotising skin lesions.^5^ Cocaine is also associated with formication, which can drive compulsive scratching and excoriation, particularly in individuals with underlying psychotic or somatic delusional beliefs.¹

**Conclusion:**

Cocaine-associated skin-picking can result in severe and enduring facial injury when driven by somatic delusional beliefs and substance-induced perceptual disturbances. This case underscores the importance of coordinated psychiatric, psychological, substance misuse, and physical healthcare interventions to prevent further harm.

